# Association between socio-economic deprivation and AHRQ composite indicator during pandemic

**DOI:** 10.1093/eurpub/ckac129.154

**Published:** 2022-10-25

**Authors:** F Cedrone, P Di Giovanni, G Di Martino, F Romano, T Staniscia

**Affiliations:** 1 Department of Medicine and Ageing Sciences, “G. d’Annunzio” University of Chieti-Pescara, Chieti, Italy; 2 Health Management of “SS. Spirito” Hospital, Local Health Authority of Pescara, Pescara, Italy; 3 Department of Pharmacy, “G. d’Annunzio” University of Chieti-Pescara, Chieti, Italy; 4 Unit of Hygiene Epidemiology & Public Health, Local Health Authority of Pescara, Pescara, Italy; 5 Department of Infectious Diseases and Public Health, “La Sapienza” University of Rome, Rome, Italy

## Abstract

**Background:**

The Agency for Healthcare Research and Quality (AHRQ) identifies preventable hospitalizations as proxy of potentially low-quality of care. Previous studies showed as socio-economic status was associated to poor diseases outcomes and to the performance of health services. Recently a particular attention was focused on the effect of the pandemic on this context. The aim of this research is to analyze the association between poor quality of primary care and socio-economic status before and during the pandemic.

**Methods:**

A retrospective observational study was conducted in Abruzzo Region, Italy. Hospital discharge records (HDR) of two different periods were selected: from April to December for 2019 and 2020. The aggregate Prevention Quality Indicator 90 (PQI-90) has been coded according to the indications of the AHRQ. The Italian socioeconomic deprivation index (DI), divided in quintiles (from 1st less deprived to the 5th most deprived) was attributed to all patient, based on the municipality of residence. A multivariate logistic regression model was performed to evaluate the association between PQI-90 and DI.

**Results:**

Totally were analyzed 253,063 HDR, of which 14,845 attributable to the PQI-90. By correcting for gender, age and number of comorbidities, the DI was not associated with the PQI-90 during 2019. During 2020 the PQI-90 was associated to 4th DI quintile (aOR 1.19;95%CI 1.09-1.30) and 5th DI quintile (aOR 1.13; 95%CI 1.03-1.23), compared to the 1st quintile.

**Conclusions:**

The impact of the pandemic on primary care has been substantial. Compared to the pre-pandemic era, during the pandemic, an association between potentially poor quality of care and the most disadvantaged socio-economic areas has been shown in Abruzzo. This evidence must be an interesting starting point for health planning in order to fight against inequalities in health services access.

**Key messages:**

• The reduction in the primary care quality during pandemic caused potentially preventable hospitalizations and it was associated to socioeconomic deprivation.

• The analysis of hospital admission linked to context indicators such as the deprivation index, can be a useful tool for the policy maker in order to reduce healthcare inequalities.

